# Physics of virus transmission by speaking droplets

**DOI:** 10.1073/pnas.2011889117

**Published:** 2020-09-24

**Authors:** Roland R. Netz, William A. Eaton

**Affiliations:** ^a^Fachbereich Physik, Freie Universität Berlin, 14195 Berlin, Germany;; ^b^Laboratory of Chemical Physics, National Institute of Diabetes and Digestive and Kidney Diseases, National Institutes of Health, Bethesda, MD 20892

**Keywords:** COVID-19, virus transmission, speaking droplets, SARS-CoV-2

## Abstract

To make the physics of person-to-person virus transmission from emitted droplets of oral fluid while speaking easily understood, we present simple and transparent algebraic equations that capture the essential physics of the problem. Calculations with these equations provide a straightforward way of determining whether emitted droplets remain airborne or rapidly fall to the ground, after accounting for the decrease in droplet size from water evaporation. At a relative humidity of 50%, for example, droplets with initial radii larger than about 50 μm rapidly fall to the ground, while smaller, potentially virus-containing droplets shrink in size from water evaporation and remain airborne for many minutes. Estimates of airborne virion emission rates while speaking strongly support the proposal that mouth coverings can help contain the COVID-19 pandemic.

The physics of water droplets is a well-studied subject, and its relevance to virus transmission is long known (1–10). It is a subject that has aroused renewed interest because of the COVID-19 pandemic and has motivated scientists to perform new kinds of experiments. Recently published laser light-scattering experiments of Anfinrud and coworkers (11, 12) show that the number of oral fluid droplets emitted into the air while speaking is orders of magnitude larger than previously detected using less sensitive methods (7) and that blocking such droplets is easily accomplished with a cloth mouth cover (11, 13). Previous physics calculations of droplet evaporation to determine whether droplets containing viruses remain floating in air or rapidly fall to the ground typically involve numerical simulations, which hide the fundamental mechanisms (4). In addition, the mathematics employed is too complex to be understood by other than physical scientists. We have investigated various aspects of this problem and present simple and transparent algebraic equations that capture the essential physics. Derivations of all equations are given in ref. 14.

## Results and Discussion

Our equations answer two important questions. First, how long does it take for a virus-containing droplet of a given size to fall to the ground by gravity to potentially contaminate a surface? Second, for a given relative humidity, how much time does it take for water evaporation to reduce a virus-containing droplet to a size that leaves it floating in air for a sufficiently long time to allow direct transmission of the virus to another person? The answer to the first question is easily obtained by simply equating the gravitational and Stokesian viscous forces on a falling object (*mg* = 6*πηRv*) to obtain the terminal velocity (*v*). This simplistic treatment must be justified and is given in ref. 14. The mean time for a particle to reach the ground is

$$\tau_{sed}=\frac{9\eta z_{0}}{2\rho R^{2}g}=\phi \frac{z_{0}}{R^{2}},$$ [1]

where *τ*_*sed*_ is the mean time for a droplet of radius *R* to reach the ground from a height, *z*_0_, with both *R* and *z*_0_ in units of micrometers. The prefactor, *ϕ* = 9*η*/(2*ρg*) *=* 0.85 × 10^−2^ μm∙s, is calculated from the viscosity of air at 25 °C, *η* = 1.86 × 10^−8^ g⋅μm^−1^⋅s^−1^, water density *ρ* = 10^−12^ g/μm^3^, and the gravitational constant *g* = 9.8 × 10^6^ μm/s^2^. A few examples are instructive. In the absence of water evaporation, droplets placed initially at *z*_*0*_ *=* 1.5 m (the average height above ground for the mouth of a standing human adult) with radii of 1, 10, or 100 μm will require 1.3 × 10^4^ s (∼3.5 h), 130 s, and 1.3 s, respectively, to fall to the ground.

Whether or not a virus-containing droplet will remain airborne to cause an infection requires determination of the rate of evaporation of water, which is defined by the diffusion equation in terms of the water vapor concentration profile outside of the spherical droplet. The most important effect to consider in the size regime of interest is the cooling of the droplet from the heat loss due to water evaporation, which can be determined by solving the coupled heat flux and water diffusion equations and slows down evaporation (14). The osmotic effect of nonvolatile droplet contents further decreases the evaporation rate by reducing the water vapor pressure at the droplet surface (14).

There are three different size regimes that require different theoretical treatments (14): droplet radii *R* < 70 nm, 70 nm < *R* < 60 μm, and *R* > 60 μm. We can ignore consideration of droplets with *R* < 70 nm because they are in the size regime of single virions (*τ*_*sed*_ = several days), which are not emitted without a surrounding layer of oral fluid. Droplets larger than 60 μm fall rapidly to the ground, so are of less concern here. They are dealt with theoretically elsewhere (14). We shall, therefore, only be concerned with the regime 70 nm < *R* < 60 μm. In the following, we assume that the droplet has escaped from any surrounding water vapor cloud (6) to be in ambient air.

The time (*τ*_*ev*_) it takes for complete evaporation of a pure water droplet of initial radius *R*_0_, including cooling, is

$$\tau_{ev}=\frac{R_{0}^{2}}{\theta \left(1-RH\right)},$$ [2]

where *RH* is the relative humidity, *θ* = 2*αD*_*w*_*c*_*g*_*v*_*w*_ = 4.2 × 10^2^ μm^2^/s at 25 °C is a constant with units of diffusion, and the numerical prefactor, *α* = 0.36, accounts for evaporation cooling effects (14). The diffusion constant for a water molecule in air, *D*_*w*_, is 2.5 × 10^7^ μm^2^/s, the water number concentration, *c*_*g*_, in saturated air is 7.7 × 10^5^ μm^−3^, and the water molecular volume, *v*_*w*_, in liquid water is 3.0 × 10^−11^ μm^3^, all at 25 °C.

The theory is more complex for inclusion of the osmotic effect of the nonvolatile contents of a droplet, the so-called droplet nucleus. In this case, the mean time for a droplet of initial radius *R*_0_ to shrink to a radius *R* from water evaporation is given by

$$t\left(R\right)/\tau_{ev}=1-\frac{R^{2}}{R_{0}^{2}}-\frac{2R_{dn}^{2}}{3R_{0}^{2}}\ln\left(\frac{R_{0}\left(R-R_{dn}\right)}{R\left(R_{0}-R_{dn}\right)}\right),$$ [3]

where $R_{dn}$ is the equilibrium droplet nucleus radius, which, based on a solute volume fraction for saliva of 0.03, is estimated to be ∼*R*_0_/3. The last term in Eq. **3** accounts for the vapor pressure reduction due to solutes. At *R* $\leq 1.5\;R_{dn}$, the evaporation time enters the solute-dominated regime and diverges, albeit only logarithmically, in the limit $R\to R_{dn}$. Therefore, for times prior to achieving perfect equilibrium, the logarithmic term is small enough to be neglected, and Eq. **3** simplifies to

$$t\left(R_{dn}\right)\approx \frac{R_{0}^{2}-R_{dn}^{2}}{\theta \left(1-RH\right)}.$$ [4]

At a relative humidity of *RH* = 0.5, a common value for room air, the mean evaporation times for droplets with initial radii, *R*_0_, of 1, 10, and 100 μm are 4.2 ms, 0.42 s, and 42 s, while the corresponding sedimentation mean times, *τ*_*sed*_*,* from Eq. **1** are 1.3 × 10^4^ s, 130 s, and 1.3 s. Consequently, the 1- and 10-μm droplets will dry out and stay floating for even longer, which will be determined by the radius of the droplet nuclei, *R*_*dn*_. Thus, droplets with an initial radius of *R*_o_ = 20 μm will shrink to a droplet nucleus radius of ∼7 μm in $t\left(R_{dn}\right)\approx 1.7\;s$ (Eq. **4**), with the droplet nuclei remaining airborne for about 4 min (Eq. **1**).

It is useful to define a “critical radius,” $R_{0}^{crit}$, where the evaporation and settling times are equal, that is, *t*(*R*_*dn*_) = τ_*sed*_. $R_{0}^{crit}$ is obtained by combining Eqs. **1** and **4** (with *R*_*dn*_ = *R*_0_/3) to give

$$R_{0}^{crit}={\left[1.1\phi \theta z_{0}\left(1-RH\right)\right]}^{1/4}.$$ [5]

For *RH* = 0.5 and *z*_0_ = 1.5 m, the critical radius is 42 μm. This means that droplets with radii greater than 42 μm will fall to the ground before drying out, while droplets with radii less than 42 μm will remain floating in the air in a dry state. A more accurate value for the critical radius of ∼50 μm is obtained by solving equations that take into account evaporation of droplets while sedimenting (14).

Can we say anything useful about the number of emitted virions while speaking? Table 1 shows the calculated values for initial droplet radii (*R*_0_) from 1 μm to 40 μm, using previously determined droplet production rates while speaking and saliva virion concentrations, which predicts that the number of emitted virions per minute while continuously speaking ranges from 3 to ∼2 × 10^5^. Comparing the evaporation times at a relative humidity of 50% with the sedimentation times in Table 1 shows that, for all radii in this range, droplet nuclei remain airborne for times sufficiently long that their airborne lifetime will be determined by the turnover time of the air handling system (see ref. 14). It is not known what fraction of the virions in these concentration measurements are infectious, but it has been argued that, in some systems, as few as a single active virion can cause an infection (15). The very large range of virion emission rates in Table 1 calls for both an accurate determination of the fraction of airborne virions that are infectious and accurate droplet size distributions at the high rate of emission determined by laser light scattering (11, 12).

**Table 1.** Theoretical virion emission rates (*k*), evaporation times (*t*(*R*_*dn*_)), and sedimentation times (*τ*_*sed*_) for initial radii (*R*_0_) and for droplet nuclei radii (*R*_*dn*_ = *R*_0_/3), all for *z*_0_ = 1.5 m, 25 °C, and 50% relative humidity (*RH* = 0.5)

*R*_0_ (μm)	k* (virions per min)	*t*(*R*_*dn*_)^†^ (min)	*τ*_*sed*_(droplet, *R*_*0*_)^‡^ (min)	*τ*_*sed*_(droplet nuclei,*R*_0_/3)^‡^ (min)
1	3	7 × 10^−5^	200	2 x 10^3^
3	80	6 × 10^−4^	20	200
5	400	2 × 10^−3^	8	80
10	3 × 10^3^	7 × 10^−3^	2	20
20	2 × 10^4^	3 × 10^−2^	0.5	5
40	2 × 10^5^	0.1	0.1	1

* Calculated from *k* = (4/3) π $R_{0}^{3}$ ab, *a *≈ 10^5^ droplets per min (11, 12), average *b* = 7 x 10^−6^ virions per μm^3^ (maximum *b* = 2.35 × 10^−3^ virions per μm^3^) (16).

^†^ Calculated from *t*(*R*_*dn*_) ≈ 7 x 10^−5^ $R_{0}^{2}$ min.

‡ Calculated from *τ*_*sed*_ = 210 min/*R*^2^.

Overall, the above analysis strongly supports the concept that simply speaking can be a major mechanism of person-to-person COVID-19 transmission and that covering the mouth in public, as suggested by the work of Anfinrud and coworkers (11–13) and others (10, 17), could help to more rapidly contain and potentially end the pandemic.

## Data Availability

All study data are included in the article.
